# Evolution of the Structure and Morphology of Dual-Linker ZIF-301-eIm

**DOI:** 10.3390/molecules29143395

**Published:** 2024-07-19

**Authors:** Ping Wei, Boyao Xie, Jiang Wang, Yanjun Wu, Qi Shi, Jinxiang Dong

**Affiliations:** Shanxi Key Laboratory of Chemical Product Engineering, College of Chemical Engineering and Technology, Taiyuan University of Technology, Taiyuan 030024, China; 17835202570@163.com (P.W.); 15034577676@163.com (B.X.); jwang47@163.com (J.W.); 13182300933@163.com (Y.W.); dongjinxiangwork@hotmail.com (J.D.)

**Keywords:** zeolite imidazolate frameworks, CHA topology, acetone and butanol separation

## Abstract

Few studies have reported on the continuous evolution of dual-linker zeolitic imidazolate frameworks’ (ZIFs) structure and morphology during the crystal growth process. Herein, we report the synthesis of a novel ZIF material with CHA topology (ZIF-301-eIm) via the combination of a small-sized 2-ethylimidazole (eIm) with the large-sized 5-chlorobenzimidazole ligand. A series of derivative materials with distinct structures and morphologies were obtained via two pathways: (1) insufficient amount of eIm with prolonged crystallization time (pathway A) and (2) sufficient amount of eIm with prolonged crystallization time (pathway B). Various characterization techniques revealed the continuous evolution of structure and morphology during the crystal growth process. Insufficient amount of eIm and crystallization time (crystallization pathway A) led to ZIF-301-eIm derivatives with defective and open structures alongside an aggregated morphology of nanoparticles. Prolonging the crystallization time allowed small-sized eIm ligands to gradually fill into the framework, resulting in the formation of ZIF-301-eIm-A5 characterized by complete but dense structures with a perfect polyhedral morphology. Remarkably, a sufficient amount of eIm during synthesis (crystallization pathway B) formed ZIF-301-eIm-B1 with a similar structure and morphology to ZIF-301-eIm-A5 in just 1 day. ZIF-301-eIm-B3, with intact, dense structures, exhibits superior acetone/butanol separation performance compared to ZIF-301-eIm-A3 due to small pore windows and large cages facilitating selective adsorption of acetone through exclusion separation.

## 1. Introduction

Zeolitic imidazolate frameworks (ZIFs) are a subclass of nanoscale porous metal–organic frameworks (MOFs) with a zeolite-like structural topology composed of metal centers and imidazolate linkers [1,2,3,4,5]. ZIFs have significant advantages in a variety of applications, such as catalysis and adsorption separation [6,7,8,9,10,11,12,13,14]. ZIFs can be classified according to the number of imidazolate linker types involved [15] as sole-linker [16,17,18], dual-linker [19,20,21] and tri-linker structures [22]. Precise manipulation of the dual-linker chemical composition allows for the tailored adjustment of the pore surface structure and pore surface properties of ZIFs [23,24,25], resulting in their superior performance relative to sole-linker ZIFs, positioning them as highly promising materials [26,27].

In 2014, Yaghi reported three typical dual-linker ZIFs, ZIF-300 [28,29], ZIF-301 [30,31,32,33], and ZIF-302 [34,35], with specific framework compositions and CHA topology, which are based on a dual-linker synthesis of strategy utilizing the same small-sized linker, 2-methylimidazole, in conjunction with various large-sized linkers, including 5-bromobenzimidazole, 5-chlorobenzimidazole (clbIm) and 5-methylbenzimidazole [3]. Notably, CHA-type ZIFs can only be synthesized via a mixed-ligand approach [6,8]. Different large-sized ligands have been used to study the synthesis of various ZIFs with CHA topology. Indeed, exploring the substitution of 2-methylimidazole with other small-sized ligands to obtain new materials presents an intriguing question. Moreover, observations regarding the continuous evolution of the structure and morphology during the crystal growth process of dual-linker ZIFs remain an almost unexplored area to date.

In this paper, we have synthesized a novel ZIF material, ZIF-301-eIm, by introducing the small-sized 2-ethylimidazole (eIm) ligand in combination with the large-sized clbIm ligand. However, ZIF-301-eIm exhibited various dense structures, which prompted us to investigate the crystal growth process of dual-linker ZIF-301-eIm. Subsequently, a series of ZIF-301-eIm derivatives were obtained with different crystallization states via two pathways: (1) insufficient eIm with prolonged crystallization time and (2) sufficient eIm with prolonged crystallization time. Interestingly, we observed the continuous evolution of the structure (from defective and open structure to intact but dense structure) and morphology (from an aggregated morphology of nanoparticles to a perfect polyhedral appearance with a smooth surface) of the ZIF-301-eIm derivatives throughout the process. Two representative materials, one exhibiting a defective structure with an aggregated nanoparticle morphology and the other possessing an intact dense structure with a smooth polyhedral morphology, were selected for separating acetone and butanol in aqueous solutions through adsorption, revealing two distinctly opposite adsorption separation performances. Our research will enrich the understanding of the crystallization process of dual-linker ZIFs.

## 2. Results and Discussion

**Scheme 1:**
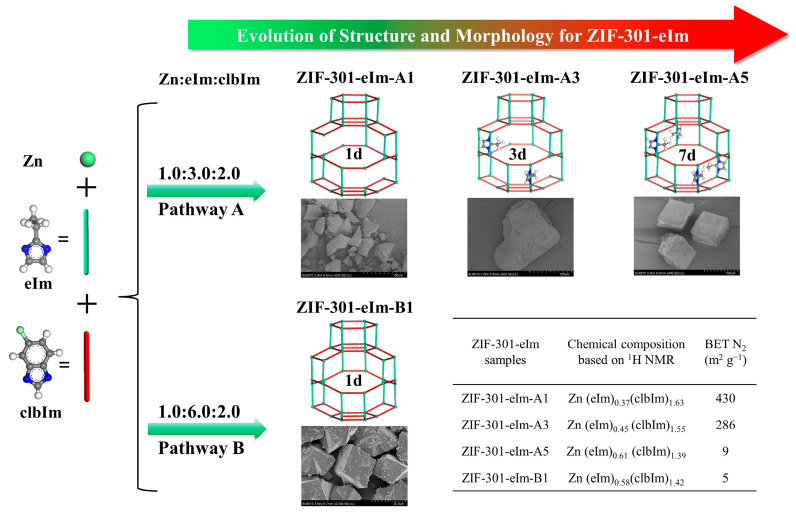
The crystal formation process of ZIF-301-eIm derivatives.

To date, there have been a few observations of the continuous evolution of the structure and morphology during the crystallization process of dual-linker ZIFs [36,37]. Our study investigated the structural and morphological changes during the evolution of dual-linker ZIF-301-eIm derivatives via two crystallization pathways achieved by extending the crystallization time and altering the feed ratio of the dual-linkers (Scheme 1). Specifically, the crystal structure evolves from a defective and open structure to a complete but dense structure, while the morphology transitions from an aggregated nanoparticle morphology to a perfect polyhedral appearance with a smooth surface. The adsorption separation performances toward acetone/butanol were explored using the ZIF-301-eIm derivatives with various crystallization states.

### 2.1. Synthesis and Characterization of ZIF-301-eIm Derivatives via Two Crystallization Pathways

Yaghi reported in 2014 that mixed-linker ZIF-301 was a CHA-type framework with a Zn(mIm)_0.94_(clbIm)_1.06_ composition [3]. Here, the CHA-type ZIF-301-eIm derivatives were first prepared using a combination of the eIm ligand and cbIm ligand, and the changes in the properties during the evolution of the ZIFs crystals, such as framework composition, pore structure, and morphology, were studied.

**Figure 1:**
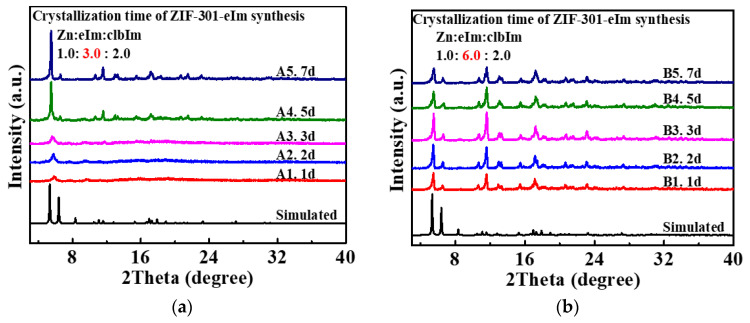
PXRD analysis of (**a**) ZIF-301-eIm-A and (**b**) ZIF-301-eIm-B derivatives obtained after different crystallization times.

By employing two crystallization pathways, which involve insufficient eIm with prolonged crystallization time and sufficient eIm with prolonged crystallization time, a range of ZIF-301-eIm derivatives with a dual-linker composition of Zn(eIm)_x_(clbIm)_2−x_ (x = 0.37–0.64) were synthesized (Table 1) and named as ZIF-301-eIm-An/Bn (where A and B represent the different crystallization pathways, and n represents the sample name). Specifically, pathway A represents a 3:2 feed ratio of eIm:clbIm, while pathway B represents a 6:2 feed ratio of eIm:clbIm with n = 1, 2, 3, 4, and 5 representing crystallization times of 1, 2, 3, 5, and 7 d, respectively. PXRD analysis (Figure 1a,b) was used to confirm the phases of ZIF-301-eIm derivatives synthesized with varying crystallization times for the two feeding ratios. The diffraction peak positions of the samples matched the peaks of ZIF-301 described in the literature (CCDC number: 995219) [3], which indicated the successful synthesis of the ZIF-301-eIm derivatives. In crystallization pathway A (Figure 1a), ZIF-301-eIm-An was synthesized using a 3:2 feed ratio of eIm:clbIm. However, only weak diffraction peaks were observed after a shorter synthesis time (<3 d), indicating the defective crystal quality. Surprisingly, when the synthesis time was extended from 3 to 5 d, there was a significant increase in the intensity of the diffraction peaks, indicating a substantial improvement in the crystal quality.

ZIF-301-eIm-Bn was obtained via crystallization pathway B (Figure 1b) using an eIm:clbIm feed ratio of 6:2. Strong diffraction peaks were observed after a much shorter crystallization time of 1 d, indicating that high-quality crystals were obtained.

**Figure 2:**
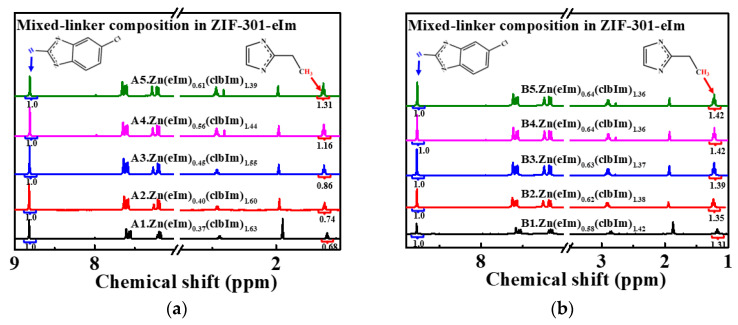
The mixed-linker compositions of the (**a**) ZIF-301-eIm-A and (**b**) ZIF-301-eIm-B derivatives based on ^1^H NMR spectroscopy.

Subsequently, the skeleton ligand composition of the ZIF-301-eIm derivatives were determined using ^1^H-NMR spectroscopy (Figures S1–S10). The skeleton composition and sample names of the relevant samples are shown in Table 1. The liquid ^1^H-NMR spectra in Figure 2a show that the content of clbIm in the skeleton of the ZIF-301-eIm-A sample was always higher than that of eIm, indicating that the large-sized clbIm ligand is preferentially doped into the ZIF-301-eIm-A skeleton due to its higher coordination affinity compared to the small-sized eIm ligand.

**Figure 3:**
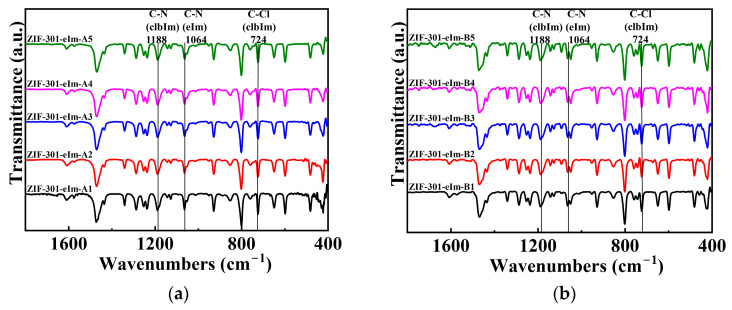
The FT-IR spectra obtained for the (**a**) ZIF-301-eIm-A and (**b**) ZIF-301-eIm-B derivatives.

Interestingly, there was a gradual augmentation in the relative content of the eIm ligands upon extending the crystallization time in crystallization pathway A, indicating that the eIm ligands were filling the framework. In crystallization pathway B, the results depicted in Figure 3b show that the composition of the two ligands within the framework remains essentially consistent for the materials synthesized using different crystallization times, i.e., ZIF-301-eIm-B1 (1 d) and ZIF-301-eIm-B5 (7 d). In addition, the proportion of eIm ligands in the framework of ZIF-301-eIm-B1 was similar to that of ZIF-301-eIm-A5, indicating that a high concentration of eIm ligands used during the synthesis facilitates their incorporation into the framework. These results suggest that both the crystallization time and the concentration of eIm ligands can influence the ratio of the dual ligand components within the framework.

Since the ZIF-301-eIm derivatives were all prepared from clbIm and eIm ligands, the materials exhibit similar FT-IR spectra, as shown in Figure 3. The peak observed at 1064 cm^−1^ was attributed to the stretching vibrations of C-N in 2-ethylimidazole. The peak at 1188 cm^−1^ corresponds to the stretching vibrations of C-N in 5-chlorobenzimidazole. The peak at 724 cm^−1^ was due to the stretching vibrations of C-Cl in 5-chlorobenzimidazole.

The TG curves show that all of the ZIF-301-eIm derivatives have high thermal stability (Figure S12a,b); no significant weight loss was observed before 400 °C, and their framework structures only began to undergo thermal decomposition and collapse after 400 °C.

### 2.2. Pore Structure Analysis of the ZIF-301-eIm Derivatives Prepared via Two Crystallization Pathways

**Figure 4:**
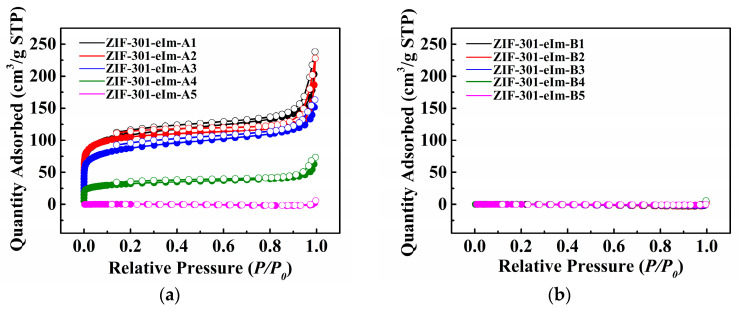
N_2_ isotherms obtained for the (**a**) ZIF-301-eIm-A and (**b**) ZIF-301-eIm-B derivatives at 77 K.

**Figure 5:**
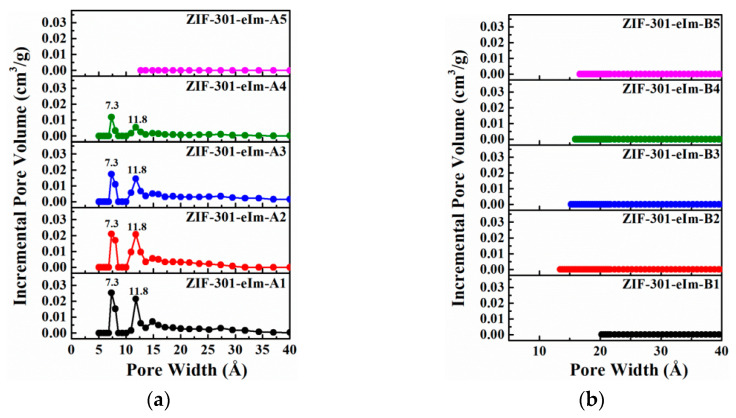
The pore size distributions of the (**a**) ZIF-301-eIm-A and (**b**) ZIF-301-eIm-B derivatives calculated based on the N_2_ isotherms at 77 K obtained using DFT calculations.

**Figure 6:**
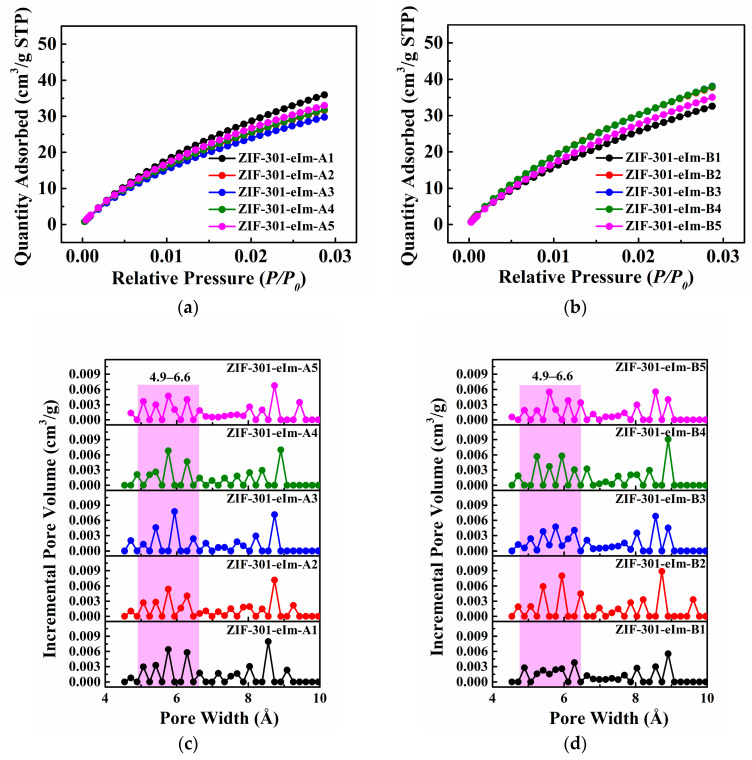
The CO_2_ isotherms obtained for the (**a**) ZIF-301-eIm-A and (**b**) ZIF-301-eIm-B derivatives at 273 K; the pore size distributions of the (**c**) ZIF-301-eIm-A and (**d**) ZIF-301-eIm-B derivatives obtained using DFT calculations.

The BET surface area and pore size distribution of ZIF-301-eIm derivatives were determined using N_2_ and CO_2_ as probe molecules (Figure 4, Figure 5 and Figure 6). In crystallization pathway A, using an eIm:clbIm feed ratio of 3.0:2.0, ZIF-301-eIm-A1, ZIF-301-eIm-A2, and ZIF-301-eIm-A3 display typical type I isotherm characteristics (Figure 4a), and their BET surface areas were determined to be 430, 363, and 286 m^2^ g^−1^ based on the N_2_ adsorption isotherm (Table 1), but with defective crystal quality based on our PXRD analysis. In addition, the pore size distribution predominantly ranged from 7.3 to 11.8 Å for ZIF-301-eIm-A1, ZIF-301-eIm-A2, and ZIF-301-eIm-A3 based on the N_2_ adsorption isotherms obtained using density functional theory (DFT) calculations (Figure 5a). Notably the BET surface area and micropore volume of the ZIF-301-eIm-An derivatives gradually decreased upon increasing the crystallization time. Specifically, the ZIF-301-eIm-A5 sharply decreased to 9 m^2^ g^−1^ after a synthesis time of 7 d (Table 1) and the micropores ranging from 7.3 to 11.8 Å were not observed (Figure 5a), but it exhibited extremely high-quality crystals based on our PXRD analysis. This phenomenon is commonly interpreted as the replacement of the small-sized eIm linker with the large-sized clbIm linker, resulting in a gradual increase in the relative proportion of clbIm in the ZIF-301-eIm framework. However, the ^1^H NMR spectra present a completely opposite phenomenon, showing a gradual increase in the relative proportion of the small-sized eIm linker in the framework. We believe that, when the amount of eIm and crystallization time are insufficient, the partial absence of the small-sized linker in the framework results in a large BET surface area. Upon prolonging the crystallization time, the missing small-sized linkers gradually fill the framework, leading to the formation of a complete but dense crystal structure. Therefore, for ZIF-301-eIm-A5, the BET value significantly reduced and the micropores within the range of 7.3–11.8 Å disappeared.

CO_2_ is a very effective probe molecule for studying small pore and dense materials [38]. Based on our CO_2_ adsorption curve obtained at 273 K, it was evident that ZIF-301-eIm-A5 remained porous to small-sized molecules. The pore size distribution predominantly ranged from 4.9 to 6.6 Å (Figure 6c) and its BET surface area showed no significant change when compared to ZIF-301-eIm-A1 (Table 1) based on our CO_2_ adsorption isotherm. The CO_2_ adsorption results demonstrate that the formation of a dense structure by filling of the framework with the small-sized eIm ligands only affects the pore size of ZIF-301-eIm and not the cage size.

In crystallization pathway B, using an eIm:clbIm feed ratio of 6.0:2.0 and reaction time of 1 d, the small-sized eIm linker composition in the framework of ZIF-301-eIm-B1 and its BET surface area, calculated based on the N_2_ adsorption isotherm, were comparable to those of ZIF-301-eIm-A5 prepared via pathway A (Table 1). In addition, the BET surface area calculated based on the CO_2_ adsorption isotherm was 242 m^2^ g^−1^ (Table 1), clearly indicating that ZIF-301-eIm-B1 remained porous. Upon extending the crystallization time, there is no significant change in the eIm linker component and BET surface area calculated based on the CO_2_ adsorption isotherm in the framework of ZIF-301-eIm-Bn. These results indicate that complete crystal structures can be obtained in a shorter crystallization time when a sufficient amount of eIm is added during the synthesis. SEM images also revealed the evolution of crystal structures during this process.

### 2.3. Morphological Analysis of the ZIF-301-eIm Derivatives Prepared via Two Crystallization Pathways

**Figure 7:**
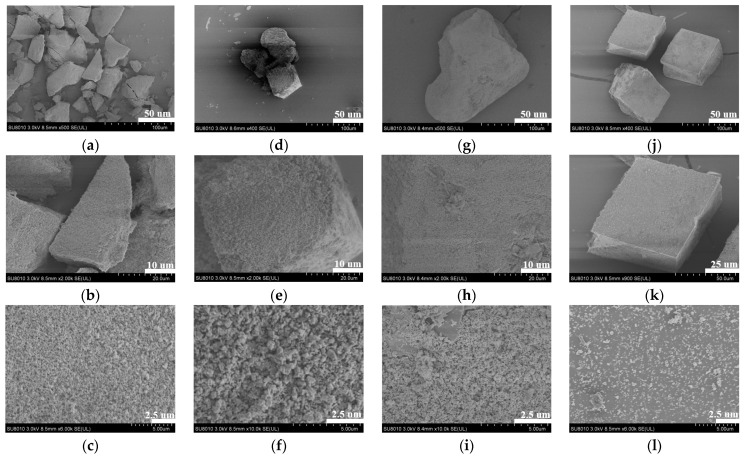
SEM images of (**a**–**c**) ZIF-301-eIm-A1, (**d**–**f**) ZIF-301-eIm-A2, (**g**–**i**) ZIF-301-eIm-A3, and (**j**–**l**) ZIF-301-A5.

**Figure 8:**
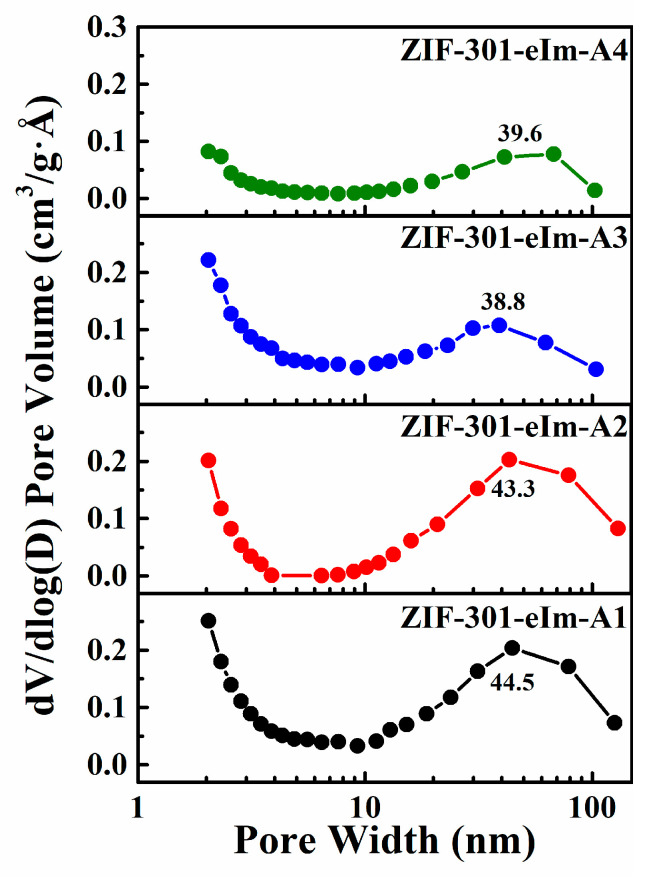
The mesoporous size distributions of the ZIF-301-eIm-A derivatives calculated based on the N_2_ isotherms obtained at 77 K using the BJH model. Note: for ZIF-301-eIm-A5, there are no valid data points when using the BJH model to calculate the mesopore size distribution.

**Figure 9:**
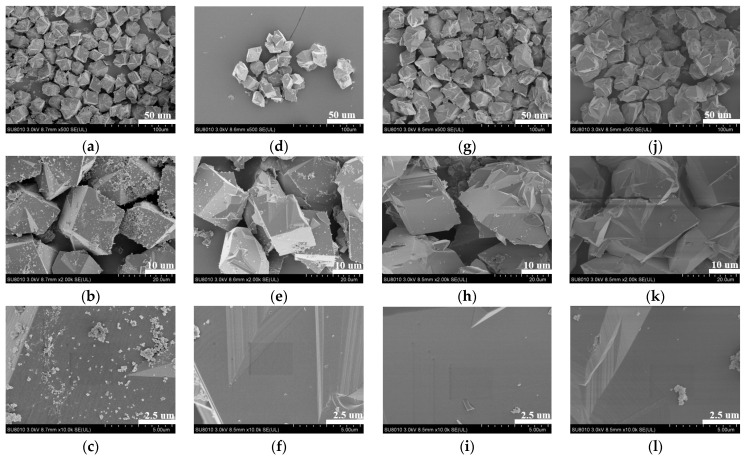
SEM images of (**a**–**c**) ZIF-301-eIm-B1, (**d**–**f**) ZIF-301-eIm-B2, (**g**–**i**) ZIF-301-eIm-B3, and (**j**–**l**) ZIF-301-eIm-B5.

The SEM images obtained at different magnifications for the crystallization pathway A are shown in Figure 7. When the feed ratio of eIm:clbIm was 3:2, the morphologies of ZIF-301-eIm-An after different crystallization times exhibited significant differences. At a crystallization time of 1 d, the appearance of ZIF-301-eIm-A1 was a rough and irregular blocky morphology formed by the aggregation of nanoparticles (Figure 7a–c). The pore size distribution obtained using a Barrett–Joyner–Halenda (BJH) pore structure analysis exhibited a peak at 44.5 nm, further confirming the aggregation of the nanoparticles (Figure 8). Upon prolonging the crystallization time, the morphology of the derivatives gradually evolved into perfect cubic shapes with a smooth surface, as observed in ZIF-301-eIm-A5 (Figure 7j–l). In addition, our BJH pore structure analysis also indicated that the volume of aggregated pores gradually decreased until they completely disappeared in ZIF-301-eIm-A5. In crystallization pathway B, when more eIm was introduced during synthesis, a remarkable perfect cubic crystal morphology was formed within an extremely short crystallization time (as short as 1 d) (Figure 9). Upon extending the crystallization time, the individual cubic particles gradually disappeared, evolving into polyhedral crystals with interlocking growth and smooth surfaces.

### 2.4. Hydrophobicity of the ZIF-301-eIm Derivatives

**Figure 10:**
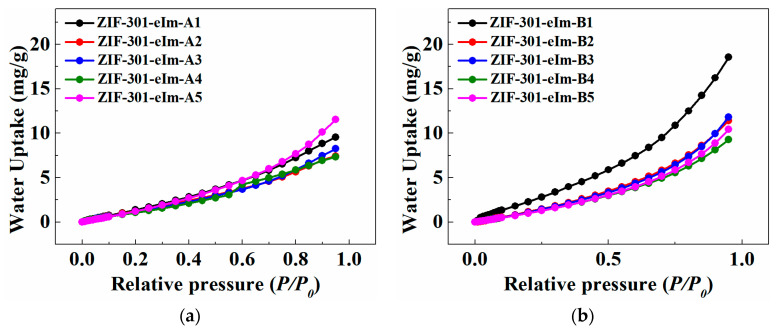
Water vapor adsorption isotherms obtained for the (**a**) ZIF-301-eIm-A and (**b**) ZIF-301-eIm-B derivatives at 25 °C.

Water vapor adsorption tests were performed at 298 K to confirm the hydrophilic/hydrophobic nature of the ZIF-301-eIm derivatives. As shown in Figure 10, the ZIF-301-eIm derivatives had a minor amount of water uptake, absorbing only a small amount of water at P/P_0_ = 0.7, and the highest water uptake was only 9.5 mg g^−1^. As a result, the ZIF-301-eIm derivatives exhibited hydrophobic properties, which are highly suitable for separating acetone and butanol from aqueous solutions.

### 2.5. Static Adsorption and Dynamic Column Adsorption of ZIF-301-eIm Derivatives toward Acetone/Butanol

To investigate the influence of the ZIF-301-eIm derivatives with varying crystallization states on the adsorption separation performance toward acetone/butanol aqueous solutions, ZIF-301-eIm-A3 with an open structure and ZIF-301-eIm-B3 with a dense structure were used for single-component static adsorption and two-component dynamic column breakthrough experiments.

**Figure 11:**
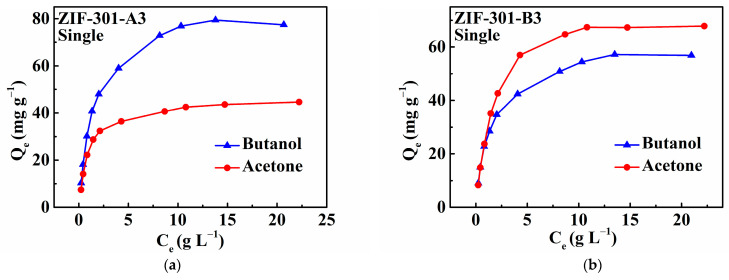
Single-component butanol and acetone isotherms obtained for (**a**) ZIF-301-eIm-A3 and (**b**) ZIF-301-eIm-B3 at 25 °C.

ZIF-301-eIm-A3 and ZIF-301-eIm-B3 were employed for single-component static adsorption experiments using acetone and butanol. Figure 11a shows that, for ZIF-301-eIm-A3 with an open structure, the adsorption capacity of the large-sized molecule butanol was higher than that of the small-sized molecule acetone. Additionally, it exhibited a saturated adsorption capacity of 44.6 mg g^−1^ for acetone and 77.4 mg g^−1^ for butanol at an initial acetone/butanol solution concentration of 20 g L^−1^ (Table S1). In contrast, the adsorption capacity of the small-sized molecule acetone was higher than that of large-sized molecule butanol for ZIF-301-eIm-B3 with a dense structure. Furthermore, it exhibited higher saturated adsorption capacities for acetone (67.9 mg g^−1^) but lower saturated adsorption capacities for butanol (56.8 mg g^−1^) at an initial acetone/butanol solution concentration of 20 g L^−1^ compared to ZIF-301-eIm-A3 with an open structure (Figure 11b). Therefore, these two samples were used to carry out two-component dynamic column breakthrough experiments in order to further investigate the adsorption separation performance of ZIF-301-eIm-A3 and ZIF-301-eIm-B3 for acetone and butanol.

**Figure 12:**
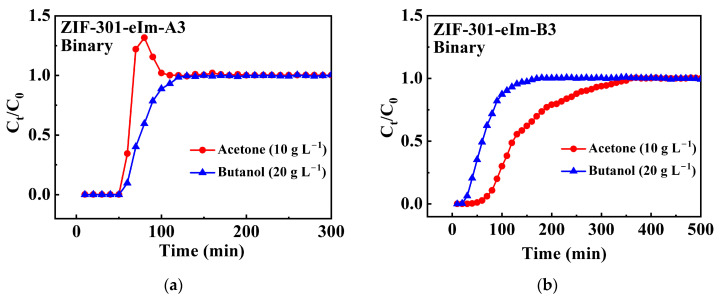
Breakthrough curves obtained for the binary–component mixture in an aqueous solution: (**a**) ZIF-301-eIm-A3 and (**b**) ZIF-301-eIm-B3 (packed column size: 250 mm × 4 mm; flow rate: 0.05 mL min^−1^; 25 °C).

The dynamic column breakthrough experiments for ZIF-301-eIm-A3 and ZIF-301-eIm-B3 still exhibited opposite separation behaviors. For ZIF-301-eIm-A3 with an open structure, it showed significant competitive adsorption phenomenon (Figure 12a), indicating that it preferentially adsorbed the large-sized molecule butanol based on thermodynamic separation. As depicted in Figure 12b, the larger molecule butanol was eluted directly from the column, while the smaller molecule acetone exhibited a broader breakthrough profile, which indicates that ZIF-301-eIm-B3 with a dense structure can achieve adsorption separation of an acetone/butanol aqueous solution to a certain extent. This was attributed to the small pore windows but large cage of ZIF-301-eIm-B3, which allows for the selective adsorption of the smaller acetone molecules via exclusion separation. Specifically, the acetone column adsorption capacity (46.9 mg g^−1^) observed for ZIF-301-eIm-B3 was greater than that of butanol (39.7 mg g^−1^) and the selectivity for acetone/butanol was 2.3 (Table S2). The CO_2_ and N_2_ static adsorption isotherms of ZIF-301-eIm-A3 and ZIF-301-eIm-B3 at 25 °C have also been investigated (Figure S16).

## 3. Materials and Methods

### 3.1. Chemicals

Zinc nitrate hexahydrate (99.0%) was obtained from Sinopharm Chemical Co., Ltd., Shanghai, China, while 2-Ethylimidazole (eIm, 98.0%) and 5-chlorobenzimidazole (clbIm, 98%) were purchased from Sa’en Chemical Technology Co., Ltd., Shanghai, China. N,N-Dimethylformamide (DMF, 99.8%), ethanol, butanol, and isobutanol (99.5%) were supplied by Aladdin Reagent Co., Ltd., Shanghai, China. Acetone came from Thermo Fisher Scientific Technology Co., Ltd., Waltham, MA, USA. All chemicals were obtained from commercial suppliers and used without further purification.

### 3.2. Preparation of ZIF-301-eIm Derivatives via Two Crystallization Pathways

ZIF-301-eIm derivatives were synthesized using two crystallization pathways. In crystallization pathway A, the ZIF-301-eIm derivatives were synthesized using a 3:2 feed ratio of eIm:clbIm using the following specific steps: A mixed solution composed of DMF (95 mL) and water (5 mL) was evenly divided into two parts. Zinc nitrate hexahydrate (3.65 mmol) was dissolved in 50 mL of DMF aqueous solution using ultrasonication. Subsequently, a mixture of 5-chlorobenzimidazole (7.3 mmol) and 2-ethylimidazole (10.95 mmol) was also dissolved in 50 mL of the DMF aqueous solution. The zinc solution was added to the mixed ligand solution with stirring. The final mixture was heated at 120 °C for 1, 2, 3, 5, and 7 d, respectively, in a PTFE-lined reaction vessel. The as-synthesized samples were washed three times with anhydrous ethanol and underwent solvent exchange with methanol, with the methanol being changed every 12 h. After solvent exchange, the samples were compressed under 4 MPa, crushed, and sieved into 20–40 mesh pellets. In the final step, the activated ZIF-301-eIm derivative adsorbents were prepared by vacuum-oven-drying the granulated samples.

In crystallization pathway B, the ZIF-301-eIm derivatives were synthesized using a 6:2 feed ratio of eIm:clbIm, i.e., the amount of clbIm remained unchanged, while the amount of eIm was increased to 21.9 mmol (2.1 g). The remaining synthesis steps were the same as those used in pathway A.

### 3.3. Characterization

PXRD patterns were recorded on an X-ray diffractometer (Rigaku, MiniFlex II, Tokyo, Japan) equipped with a Cu K_α_ radiation source (λ = 1.5418 Å). ^1^H NMR spectra were recorded on an NMR spectrometer (Bruker, Advance III 400 M, Karlsruhe, Germany). The adsorption/desorption isotherms of N_2_ (77 K) and CO_2_ (273 K) were measured on an adsorption apparatus (Micromeritics, ASAP 2020, Norcross, GA, USA). SEM images were captured with a Hitachi SU8010 scanning electron microscope. Water vapor adsorption isotherms were recorded using a DVS Resolution dynamic vapor adsorption instrument (Surface Measurement Systems, DVS Resolution, London, UK). Gas chromatography equipped with a capillary column (PC-624) was used to determine the concentration of the solution. On a thermal analyzer, thermogravimetric measurements were performed in a static air environment (Labsystems Evo, Setaram, Caluire et Cuire, France).

### 3.4. Static Batch Adsorption

The single component adsorption isotherms of the ZIF-301-eIm derivatives with varying crystallization states in acetone and butanol aqueous solutions were recorded. An amount of 50 mg of the activated sample particles was added to single component acetone or butanol aqueous solution and allowed to stand at 25 °C for 24 h. Subsequently, the concentration of the adsorbed supernatant was analyzed using a GC-2014C instrument. For the purpose of reducing experimental errors, all experiments were repeated twice. The equilibrium adsorption capacity (Q_e_) (mg g^−1^) was calculated as follows:

(1) $$Q_{e\;}=\frac{\left(C_{0}-C_{e}\right)\cdot V}{m}$$

where C_0_ and C_e_ (g L^−1^) represent the initial and equilibrium concentrations of the sample, respectively. Adsorbent sample mass (g) is represented by m (g), whereas V (mL) is the volume of the original solution.

### 3.5. Dynamic Column Adsorption

The dynamic column breakthrough performance of the ZIF-301-eIm derivatives with varying crystallization states was studied on a self-made liquid phase breakthrough instrument. In our experiments, the simulation solution concentrations of butanol and ethanol were 20 and 10 g L^−1^, respectively. The stainless-steel adsorption column (0.4 cm × 24.8 cm) was filled with the ZIF-301 derivative particles. The simulated liquid with a flow rate of 0.05 mL min^−1^ flows out from the column, and the effluent was collected and measured using gas chromatography every 10 min. The adsorption capacity of component i was calculated as follows:

(2) $$Q_{i}=\frac{{F_{i}\cdot C}_{i,0}\cdot \int_{0}^{t_{\mathrm{total}}}(1-\frac{C_{i,t}}{C_{i,0}})\mathrm{dt}}{m}$$

where, Q_i_ (mg g^−1^) is the adsorption capacity of component i, F_i_ (mL min^−1^) is the liquid flow rate, C_i,0_ (g L^−1^) is the initial simulation solution concentration, C_i,t_ (g L^−1^) is the concentration of the material after adsorption, and m (g) is the mass of the sample.

The adsorption selectivity of component i relative to j was obtained as follows:

(3) $$S_{i,j}=\frac{Q_{i}/C_{i,0}}{Q_{j}/C_{j,0}}$$

## 4. Conclusions

We have introduced the small-sized eIm ligand combined with a large-sized ligand to synthesize a novel ZIF material with CHA topology (ZIF-301-eIm). A series of ZIF-301-eIm derivatives with different crystallization states were obtained using two crystallization pathways, i.e., insufficient eIm with prolonged crystallization time (pathway A) and sufficient eIm with prolonged crystallization time (pathway B). Furthermore, various characterization techniques were employed to meticulously observe the continuous evolution of their structure and morphology. In crystallization pathway A, only ZIF-301-eIm-A1 with a defective and open structure alongside an aggregated morphology of nanoparticles was formed within a relatively short crystallization time when the amount of eIm was insufficient. Upon prolonging the crystallization time, the missing small-sized eIm ligands gradually fill into the framework, leading to the formation of ZIF-301-eIm-A5 with a complete but dense structure. Our observations were supported by the ^1^H-NMR spectra and N_2_ adsorption curves. Correspondingly, a gradual increase in the proportion of small-sized eIm ligands in the framework, and, in contrast, N_2_ adsorption isotherms at 77 K revealed a decrease in the BET surface area. The SEM images show the morphology evolved from aggregated nanoparticles to a perfect polyhedral appearance with a smooth surface. In crystallization pathway B, when a sufficient amount of small-sized eIm ligand was introduced during the synthesis, ZIF-301-eIm-B1 with a complete and dense structure characterized by smooth polyhedral morphology was formed within just 1 d. ZIF-301-eIm-B3 with an intact but dense crystal structure exhibits superior acetone/butanol adsorption separation performance when compared to ZIF-301-eIm-A3 with a defective and open crystal structure. The small pore windows but large cage possessed by ZIF-301-eIm-B3 allow for the selective adsorption of the smaller acetone molecules through exclusion separation. Our investigation will contribute to advancing researchers’ understanding of the structural and morphological changes that occur during the crystallization process of dual-linker ZIFs.

## Figures and Tables

**Table 1:** Crystallization time, dual-linker composition, BET surface area and pore volume of the prepared ZIF-301-eIm derivatives.

ZIF-301-eIm Derivative	Crystallization Time	Chemical Composition Based on ^1^H NMR Spectroscopy ^a^	BET Surface Area (m^2^ g^−1^) ^b^		Pore Volume
			N_2_	CO_2_	(cm^3^ g^−1^) ^c^
Zn:eIm:clbIm (synthesis) = 1.0:3.0:2.0					
ZIF-301-eIm-A1	1d	Zn(eIm)_0.37_(clbIm)_1.63_	430	273	0.369
ZIF-301-eIm-A2	2d	Zn(eIm)_0.40_(clbIm)_1.60_	363	235	0.353
ZIF-301-eIm-A3	3d	Zn(eIm)_0.45_(clbIm)_1.55_	286	210	0.253
ZIF-301-eIm-A4	5d	Zn(eIm)_0.56_(clbIm)_1.44_	108	229	0.113
ZIF-301-eIm-A5	7d	Zn(eIm)_0.61_(clbIm)_1.39_	9	240	0.005
Zn:eIm:clbIm (synthesis) = 1.0:6.0:2.0					
ZIF-301-eIm-B1	1d	Zn(eIm)_0.58_(clbIm)_1.42_	5	242	0.003
ZIF-301-eIm-B2	2d	Zn(eIm)_0.62_(clbIm)_1.38_	0	293	0
ZIF-301-eIm-B3	3d	Zn(eIm)_0.63_(clbIm)_1.37_	0	297	0
ZIF-301-eIm-B4	5d	Zn(eIm)_0.64_(clbIm)_1.36_	0	285	0
ZIF-301-eIm-B5	7d	Zn(eIm)_0.64_(clbIm)_1.36_	0	284	0

^a^ Determined using ^1^H NMR spectroscopy. ^b^ The BET surface areas were obtained based on N_2_ (77 K) and CO_2_ (273 K) isotherms, respectively. ^c^ The pore volume was obtained based on N_2_ (77 K) isotherms.

## Data Availability

Data are available from the corresponding authors upon request.

## Supplementary Materials

The following supporting information can be downloaded at: https://www.mdpi.com/article/10.3390/molecules29143395/s1, Figure S1–S10: Proton nuclear magnetic resonance analyses; Figure S11: PXRD characterization of ZIF-301-eIm materials; Figure S12: TG characterization of ZIF-301-eIm materials; Figures S13–S15: the stability testing of ZIF-301-eIm-A3 and ZIF-301-eIm-B3; Figure S16: the static adsorption isotherms of single-component CO_2_ and N2 for ZIF-301-eIm-A3 and ZIF-301-eIm-B3 materials at 25 °C; Table S1: static batch adsorption of single-component at 25 °C: summary of butanol and acetone adsorption capacity of ZIF-301-eIm-A3 andZIF-301-eIm-B3; Table S2: dynamic column adsorption of binary-components at 25 °C: summary of acetone/butanol column adsorption capacity and selectivity of ZIF-301-eIm-A3 and ZIF-301-eIm-B3 packed columns.
