# Supplementary material for: Evolution of the Structure and Morphology of Dual-Linker ZIF-301-eIm
Source: Molecules. 2024 Jul 19;29(14):3395. doi: 10.3390/molecules29143395 (PMC11279793; doi:10.3390/molecules29143395)
Supplement: Supplementary file 1 [file molecules-29-03395-s001.zip › molecules-3040925-supplementary.pdf]

Supplementary Information

Table of contents

Section S1: Proton nuclear magnetic resonance analyses

Section S2: PXRD, TG characterization and static adsorption isotherms of ZIF-301-eIm materials

Section S3: Summary of adsorption capacity, selectivity of batch adsorption

SectionS4: Summary of adsorption capacity, selectivity of dynamic column adsorption

Section S1: Proton nuclear magnetic resonance analyses

Each ZIF sample (ca. 50 mg) was placed in 0.5 mL of Acetic acid-OD and then sonicated for ~10 min in order to fully digest the ZIF and dissolve the link constituents. The mole ratios of the links in each ZIF were calculated based on the integrations of the identifying signals. In all samples, the peaks of Hb in cbIm and eIm linkers are not labeled because they paryially overlap with the peaks of DMF or Acetic acid-OD.

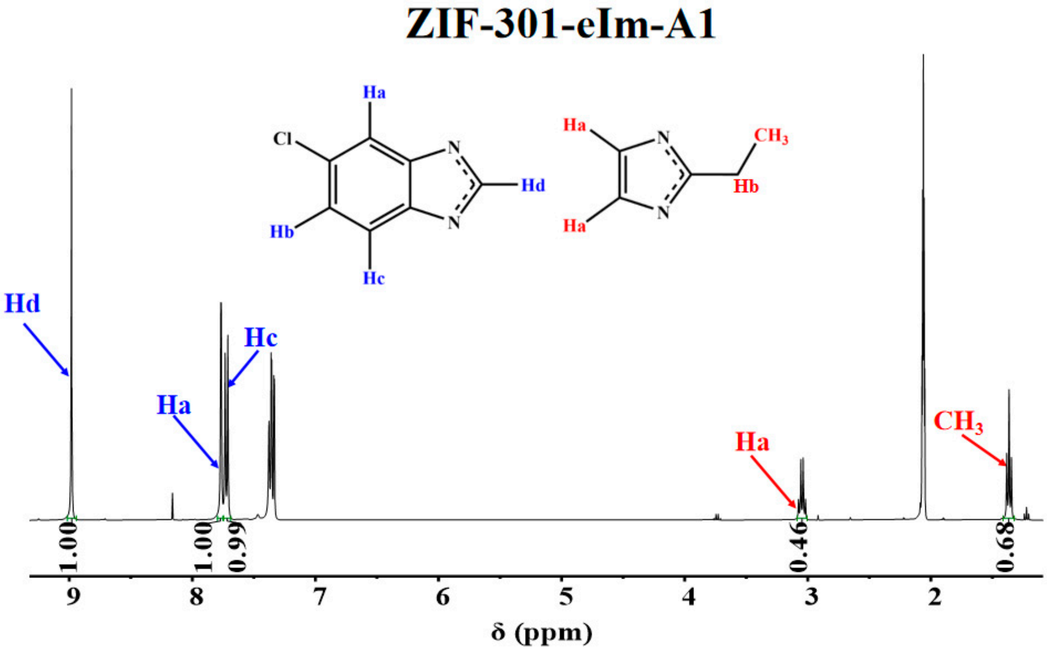

Fig. S1. ^{1}H NMR spectrum of ZIF-301-eIm-A1 post-digestion. The calculated ratio of eIm:clbIm in ZIF-301-eIm-A1 is 0.37:1.63.

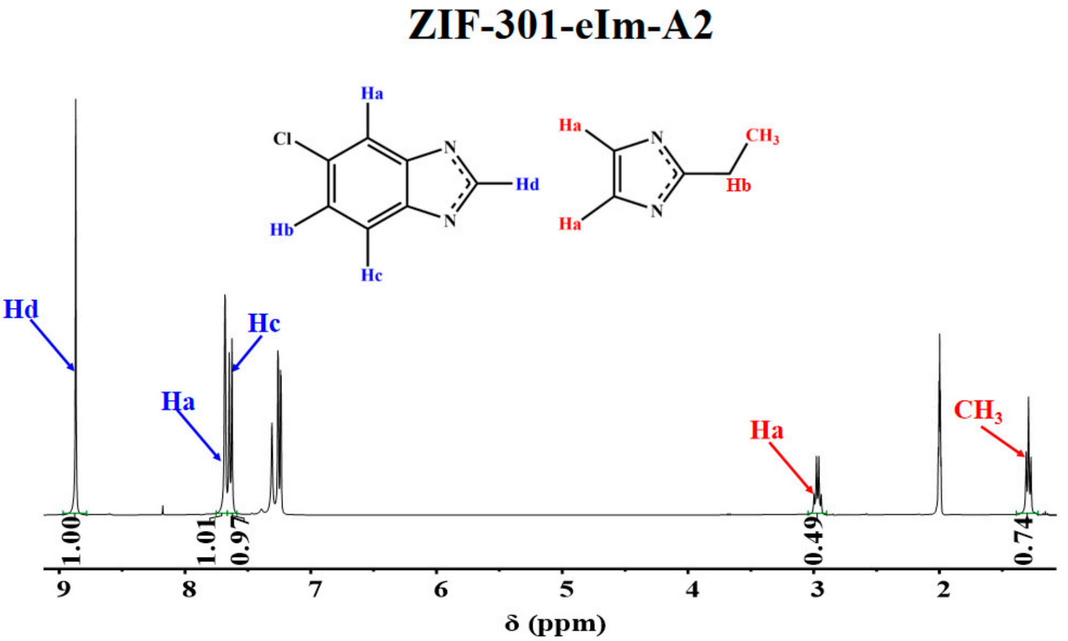

Fig. S2. ^{1}H NMR spectrum of ZIF-301-eIm-A2 post-digestion. The calculated ratio of eIm:clbIm in ZIF-301-eIm-A2 is 0.40:1.60.

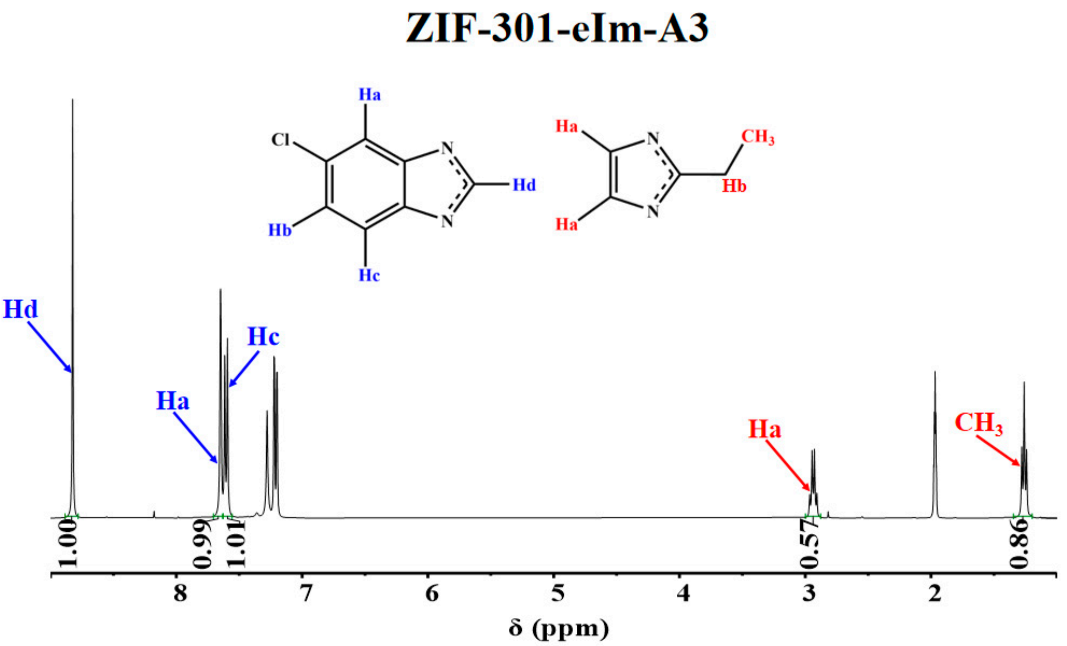

Fig. S3. ^{1}H NMR spectrum of ZIF-301-eIm-A3 post-digestion. The calculated ratio of eIm:clbIm in ZIF-301-eIm-A3 is 0.45:1.55.

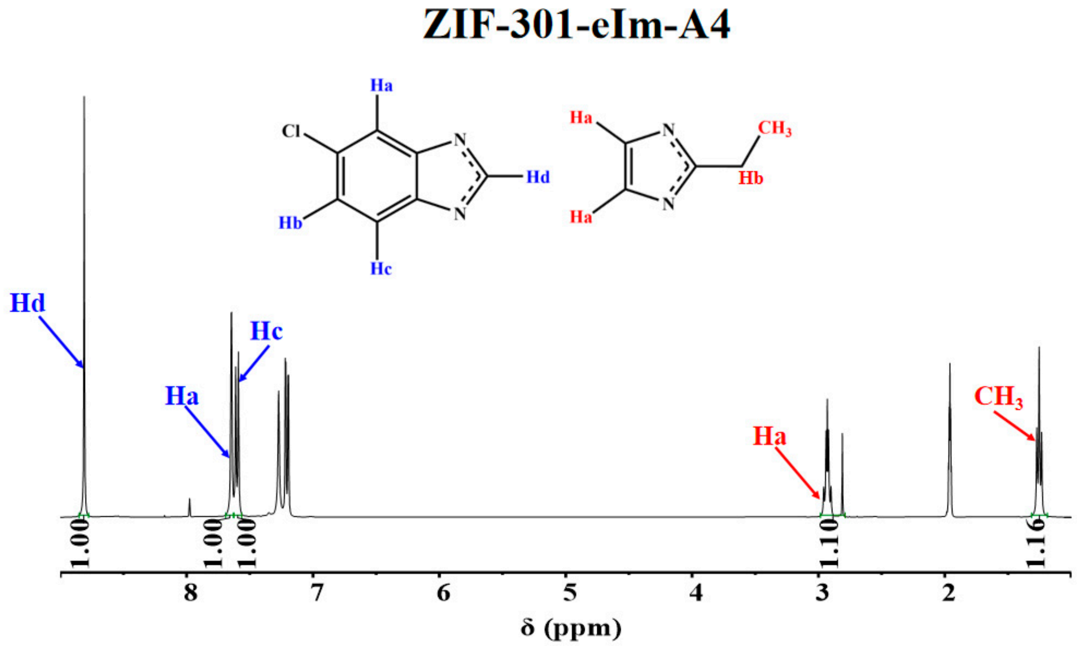

Fig. S4. ^{1}H NMR spectrum of ZIF-301-eIm-A4 post-digestion. The calculated ratio of eIm:clbIm in ZIF-301-eIm-A4 is 0.56:1.44.

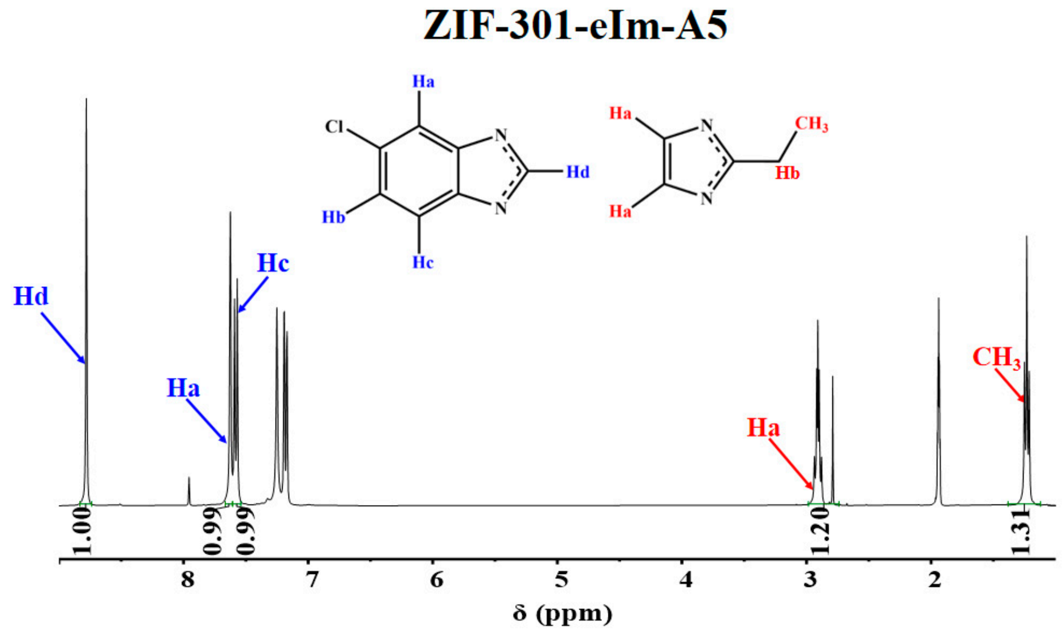

Fig. S5. ^{1}H NMR spectrum of ZIF-301-eIm-A5 post-digestion. The calculated ratio of eIm:clbIm in ZIF-301-eIm-A5 is 0.61:1.39.

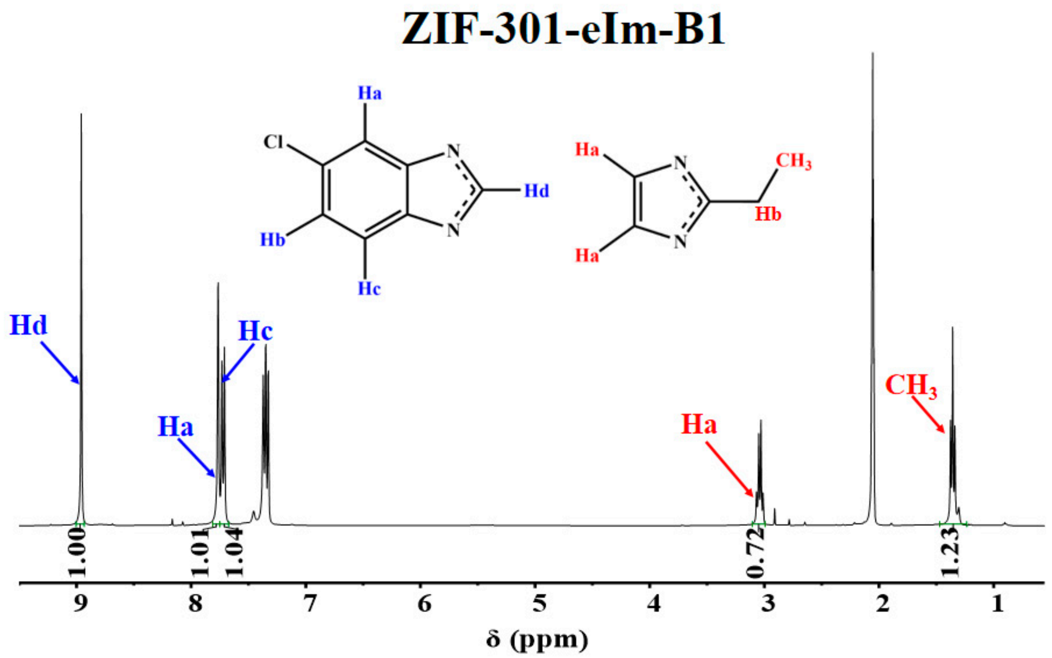

Fig. S6. ^{1}H NMR spectrum of ZIF-301-eIm-B1 post-digestion. The calculated ratio of eIm:clbIm in ZIF-301-eIm-B1 is 0.58:1.42.

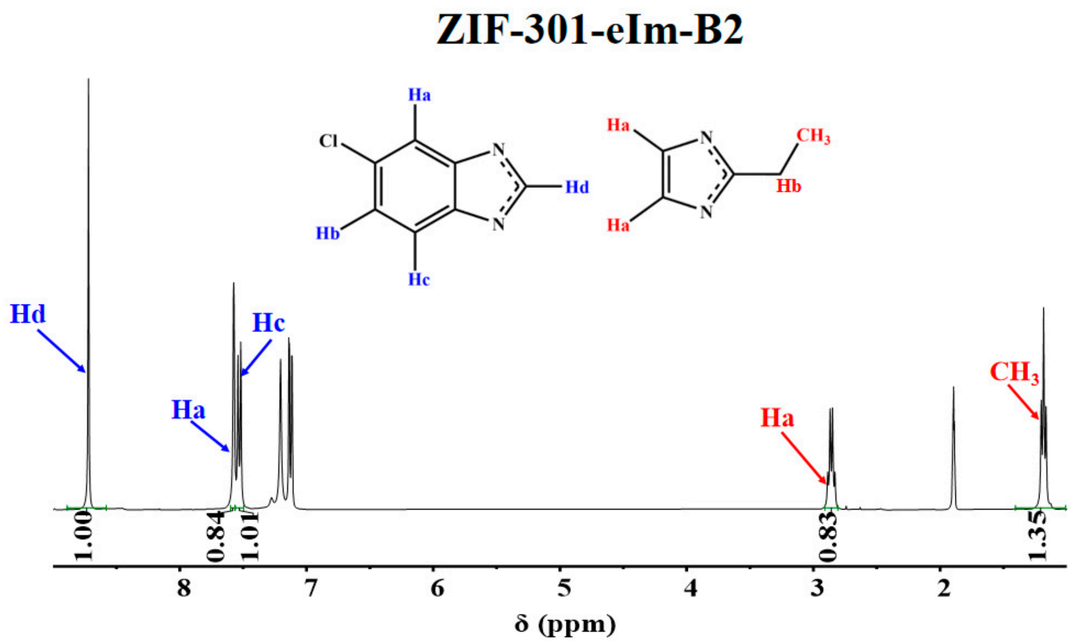

Fig. S7. ^{1}H NMR spectrum of ZIF-301-eIm-B2 post-digestion. The calculated ratio of eIm:clbIm in ZIF-301-eIm-B2 is 0.62:1.38.

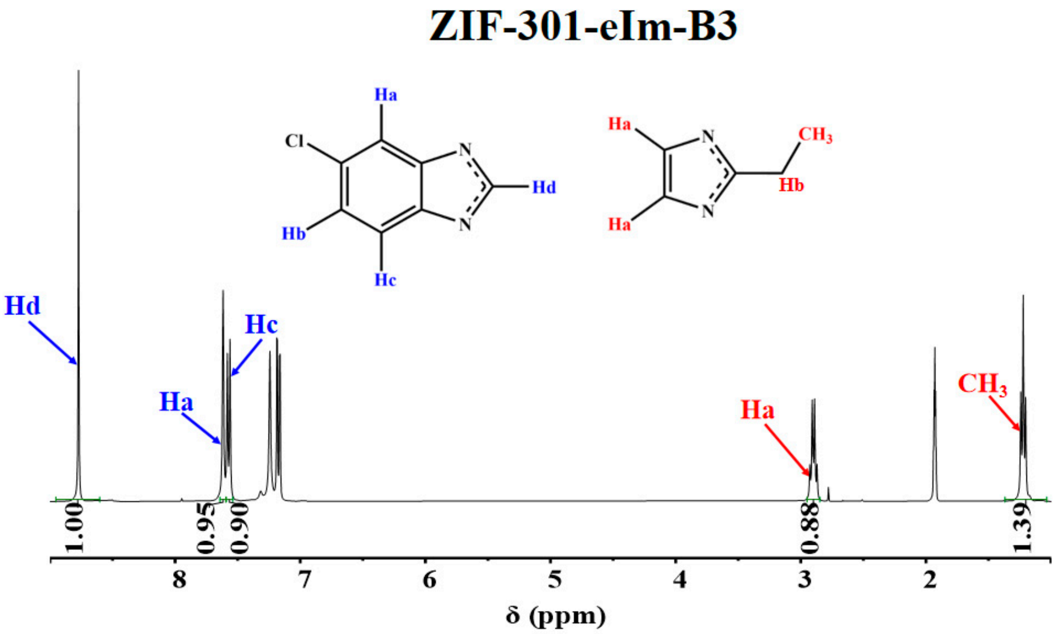

Fig. S8. ^{1}H NMR spectrum of ZIF-301-eIm-B3 post-digestion. The calculated ratio of eIm:clbIm in ZIF-301-eIm-B3 is 0.63:1.37.

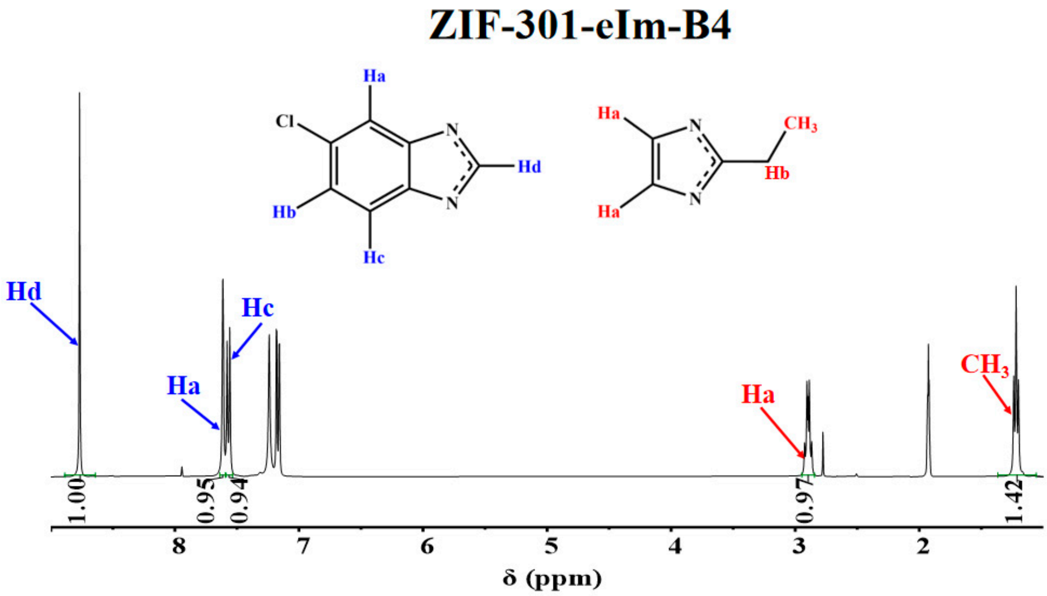

Fig. S9. ^{1}H NMR spectrum of ZIF-301-eIm-B4 post-digestion. The calculated ratio of eIm:clbIm in ZIF-301-eIm-B4 is 0.64:1.36.

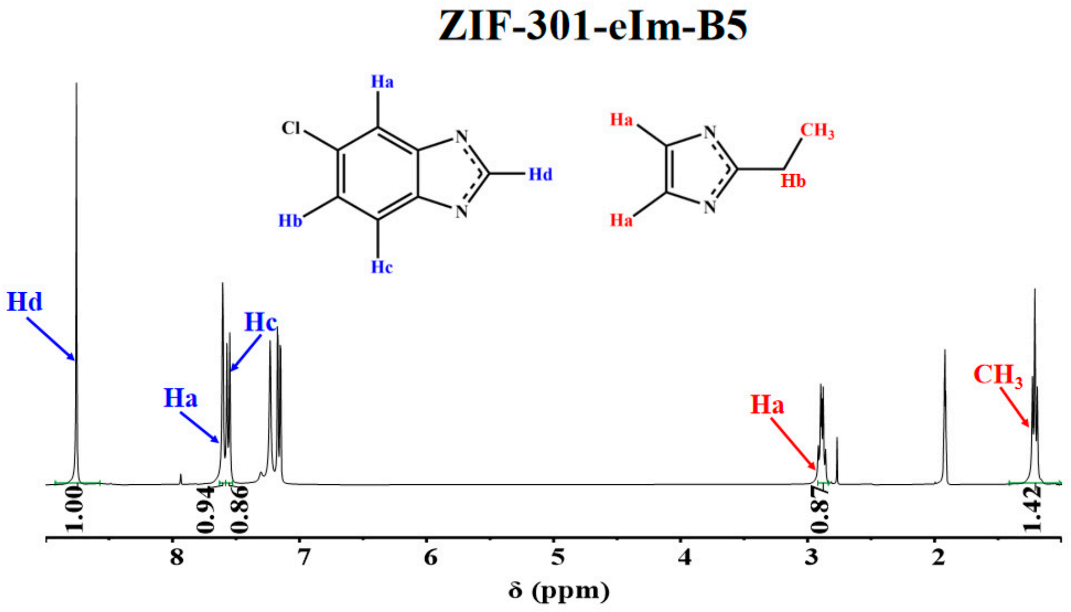

Fig. S10. ^{1}H NMR spectrum of ZIF-301-eIm-B5 post-digestion. The calculated ratio of eIm:clbIm in ZIF-301-eIm-B5 is 0.64:1.36.

Section S2: PXRD, TG characterization and static adsorption isotherms of ZIF-301-eIm materials

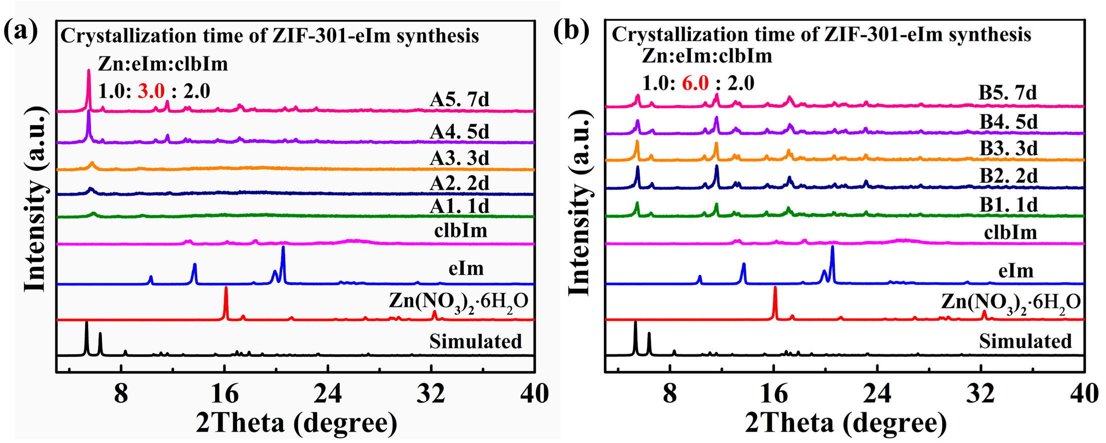

Fig. S11. PXRD analysis of (a) ZIF-301-eIm-A and (b) ZIF-301-eIm-B derivatives obtained after different crystallization times.

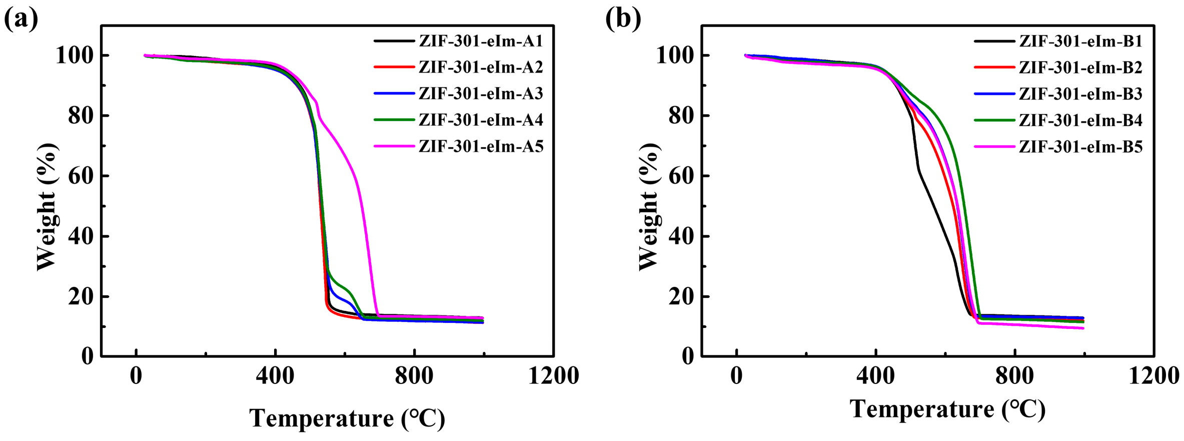

Fig. S12. (a) TG curves for ZIF-301-eIm-A1 to ZIF-301-eIm-A5 at a heating rate of 5 °C min^{-1} under air flow; (b) TG curves for ZIF-301-eIm-B1 to ZIF-301-eIm-B5 at a heating rate of 5 °C min^{-1} under air flow.

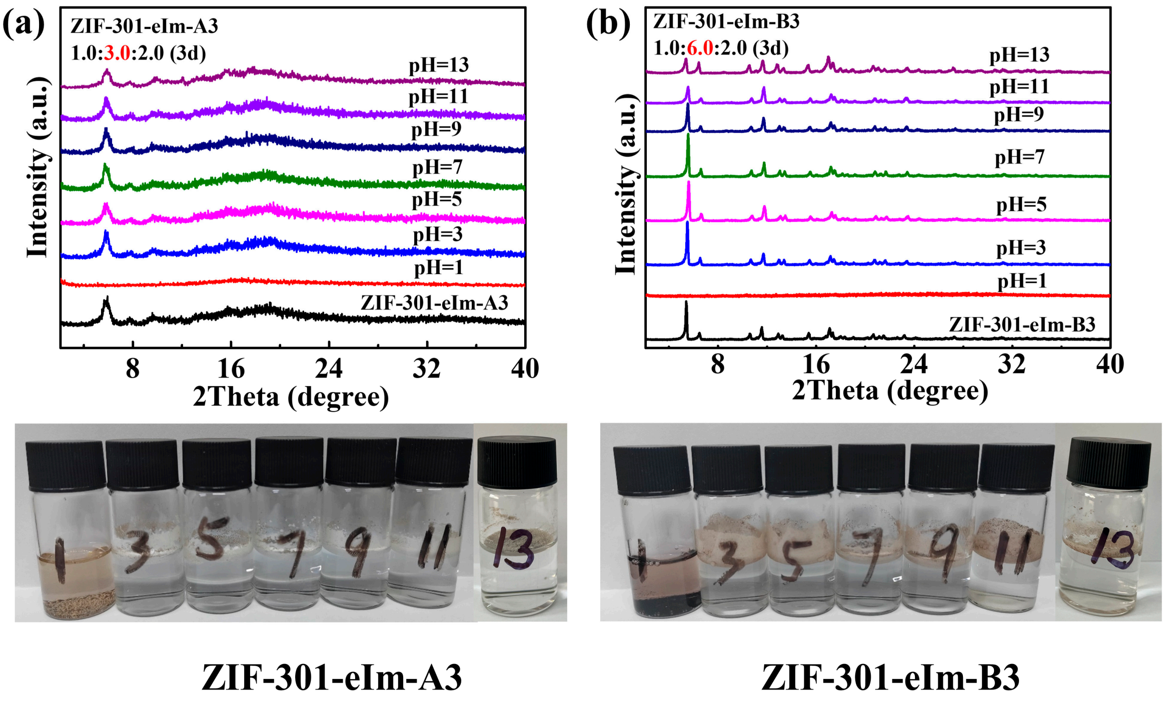

Fig. S13. The stability testing of ZIF-301-eIm-A3 and ZIF-301-eIm-B3 under various pH conditions.

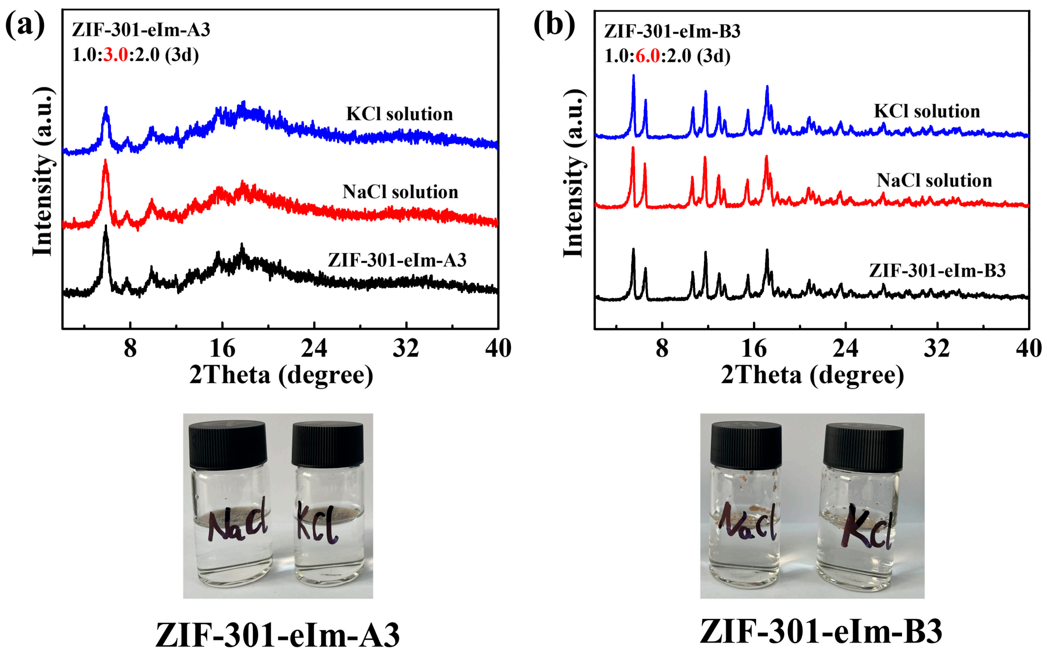

Fig. S14. The stability testing of ZIF-301-eIm-A3 and ZIF-301-eIm-B3 at different salt solutions.

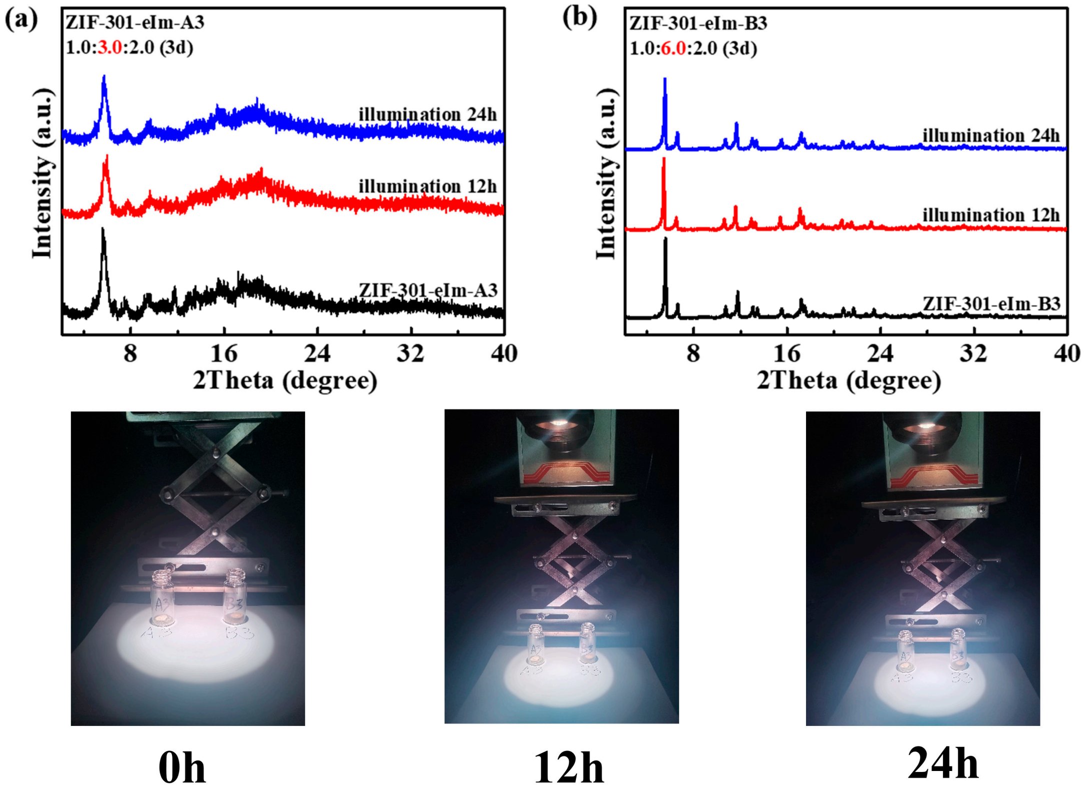

Fig. S15. Photostability testing of ZIF-301-eIm-A3 and ZIF-301-eIm-B3 materials under simulated sunlight conditions.

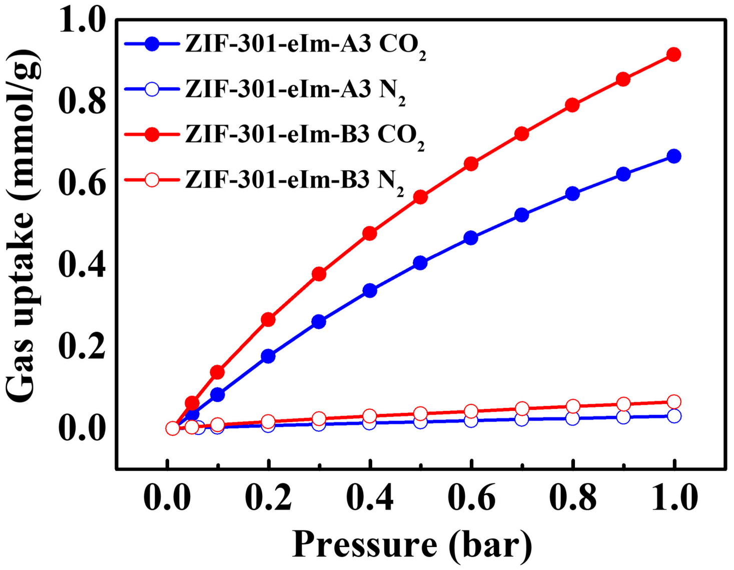

Fig. S16. The static adsorption isotherms of single-component CO_2 and N_2 for ZIF-301-eIm-A3 and ZIF-301-eIm-B3 materials at 25°C.

Section S3: Summary of adsorption capacity, selectivity of batch adsorption

Table S1. Static batch adsorption of single-component at 25 °C: summary of butanol and acetone adsorption capacity of ZIF-301-eIm-A3 andZIF-301-eIm-B3.

Adsorbent	Adsorbate	Static batch adsorption of AB single component			
		Concentration of stock solution (g L^{-1})	Adsorption amount (mg g^{-1})	Concentration of stock solution (g L^{-1})	Adsorption amount (mg g^{-1})
ZIF-301-eIm-A3	Acetone	10.1	41.8	20.6	44.6
	Butanol	19.8	77.4	19.8	77.4
ZIF-301-eIm-B3	Acetone	10.3	66.4	21.5	67.9
	Butanol	20.3	56.8	20.3	56.8

Section S4: Summary of adsorption capacity, selectivity of dynamic column adsorption

Table S2. Dynamic column adsorption of binary-components at 25 °C: summary of acetone/butanol column adsorption capacity and selectivity of ZIF-301-eIm-A3 and ZIF-301-eIm-B3 packed columns (length, 24.8 cm; diameter, 0.4 cm; at flow rate of 0.05 mL min^{-1}).

Adsorbent	Adsorbate	Dynamic column adsorption of AB binary components		
		Concentration of stock solution ($g\ L^{-1}$)	Adsorption amount ($mg\ g^{-1}$)	Acetone/Butanol selectivity
ZIF-301-eIm-A3	Acetone/Butanol	10.3/20.2	25.4/70.6	0.7
ZIF-301-eIm-B3	Acetone/Butanol	10.7/20.4	46.9/39.7	2.3
